# Woven EndoBridge treatment for anterior communicating artery aneurysm with optic neuropathy: A case report

**DOI:** 10.1016/j.radcr.2026.01.044

**Published:** 2026-02-13

**Authors:** Hideki Endo, Yasuyuki Tatsuta, Koichiro Shindo, Daishi Yamaguchi, Suguru Sakurai, Yohei Yamaguchi, Ryunosuke Yoshihara, Daigo Goto, Masahiro Okuma, Tomoaki Ishizuka, Tatsuya Ogino, Hirohiko Nakamura

**Affiliations:** a Department of Neurosurgery, Nakamura Memorial Hospital, South 1, West 14, Chuo-ku, Sapporo, Hokkaido 060-8570, Japan; b Department of Neurosurgery, Nakamura Memorial South Hospital, 2-2, Kawazoe, Minami-ku, Sapporo, Hokkaido 005-8555, Japan

**Keywords:** Anterior communicating artery, Endovascular treatment, Intracranial aneurysm, Optic nerve, Optic neuropathy, Woven EndoBridge device

## Abstract

Anterior communicating artery aneurysms rarely cause compressive optic neuropathy, and most reports describe surgical clipping or endovascular coiling. Evidence is lacking on the role of intrasaccular flow disruptors, such as the Woven EndoBridge device, in this setting. We present a 72-year-old woman with an unruptured anterior communicating artery aneurysm compressing the right optic nerve and producing an inferior visual field defect. The aneurysm was treated with Woven EndoBridge embolization, positioned to minimize mass effect. The patient’s vision improved within 1 month, and follow-up angiography confirmed complete occlusion. By demonstrating safe aneurysm occlusion and visual improvement with Woven EndoBridge deployment, our report offers a novel endovascular option where the optimal approach remains uncertain.

## Introduction

The Woven EndoBridge (WEB) device (MicroVention/Terumo, Aliso Viejo, CA, USA) is a self-expanding nitinol mesh designed to achieve aneurysm occlusion through endosaccular deployment—that is, it functions as an intrasaccular flow disruption device [1,2]. However, the treatment outcomes of WEB use in intracranial aneurysms associated with optic neuropathy remain unclear. Here, we report a case of WEB treatment for an anterior communicating artery (ACoA) aneurysm presenting with optic neuropathy.

## Case report

**Fig. 1:**
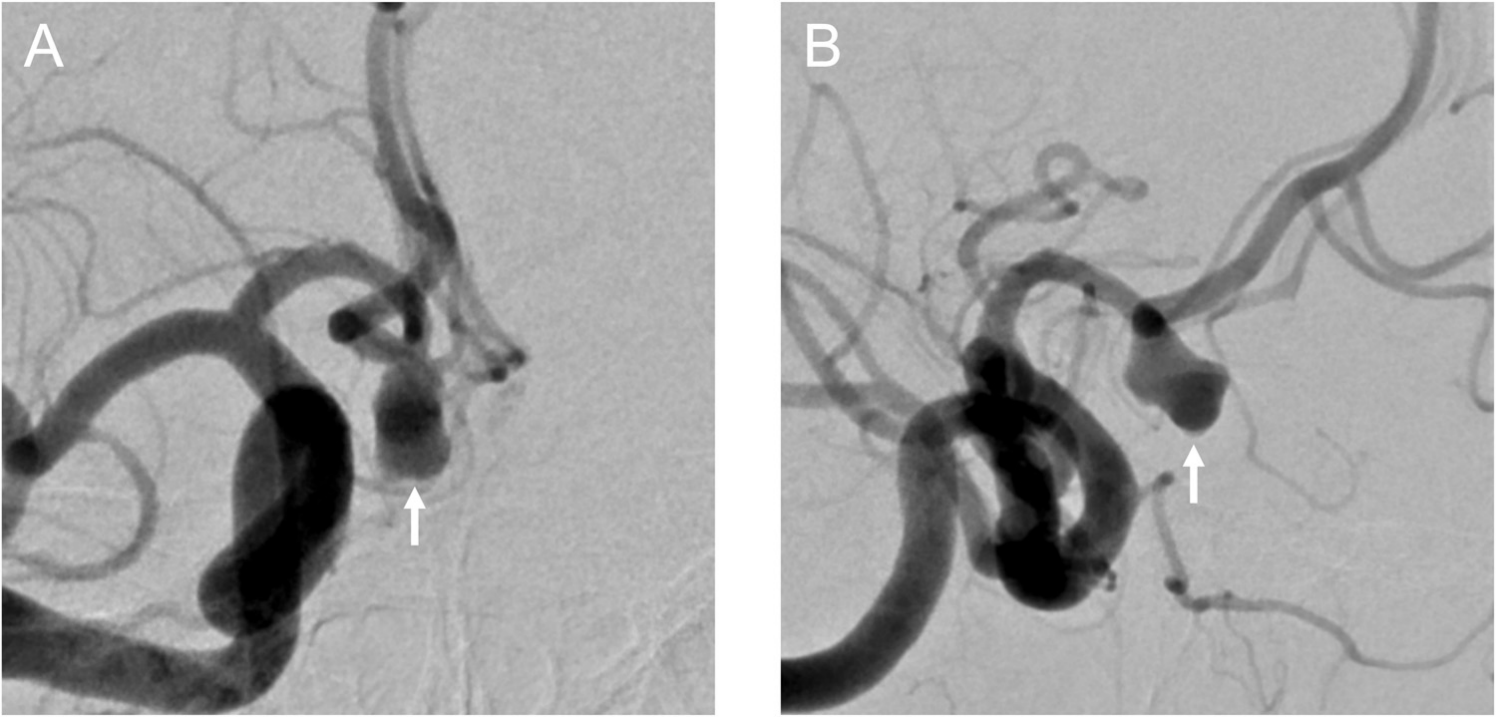
Digital subtraction angiography showing an anterior communicating artery aneurysm (arrows). (A) Anteroposterior view and (B) Lateral view.

**Fig. 2:**
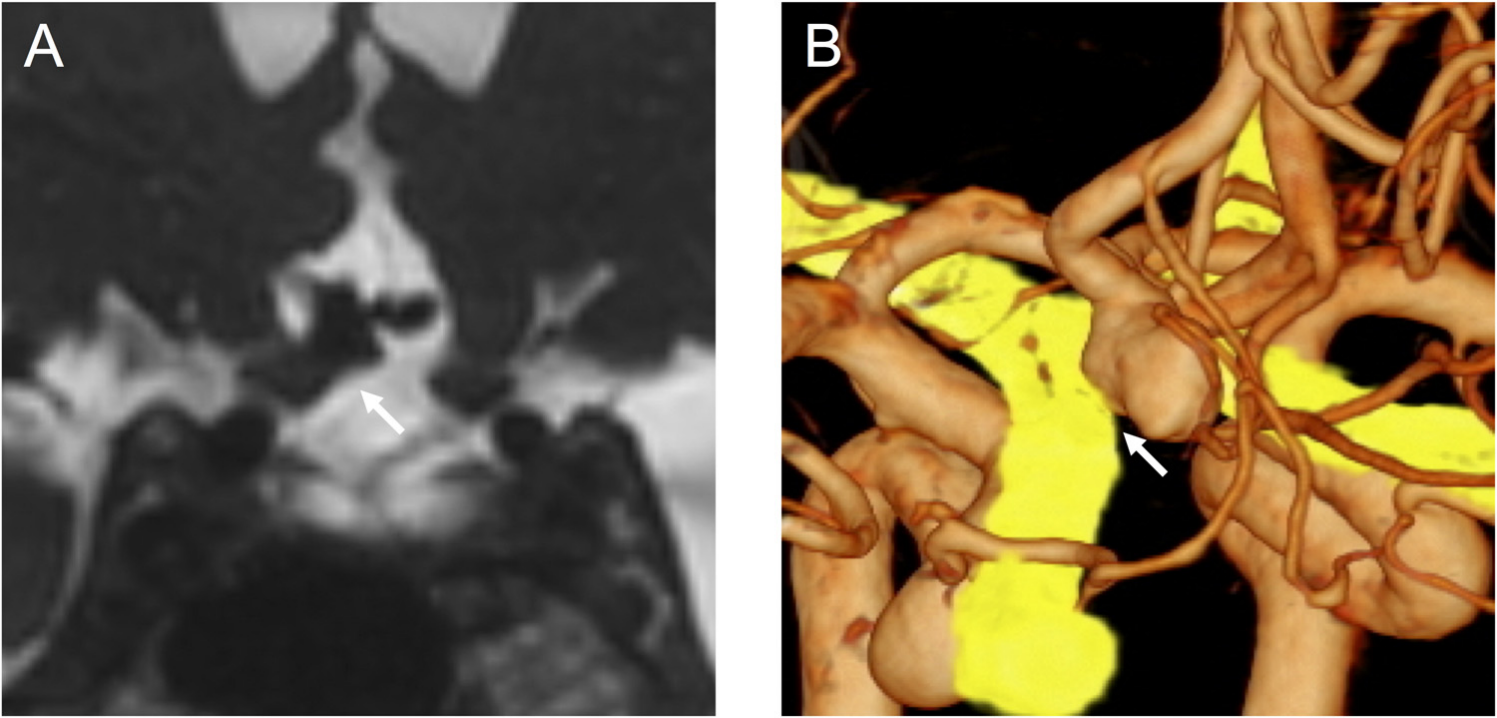
(A) Constructive interference in steady-state magnetic resonance imaging and (B) its fusion with angiography revealing that the anterior communicating artery aneurysm compressed the right optic nerve from the medial superior aspect (arrows).

**Fig. 3:**
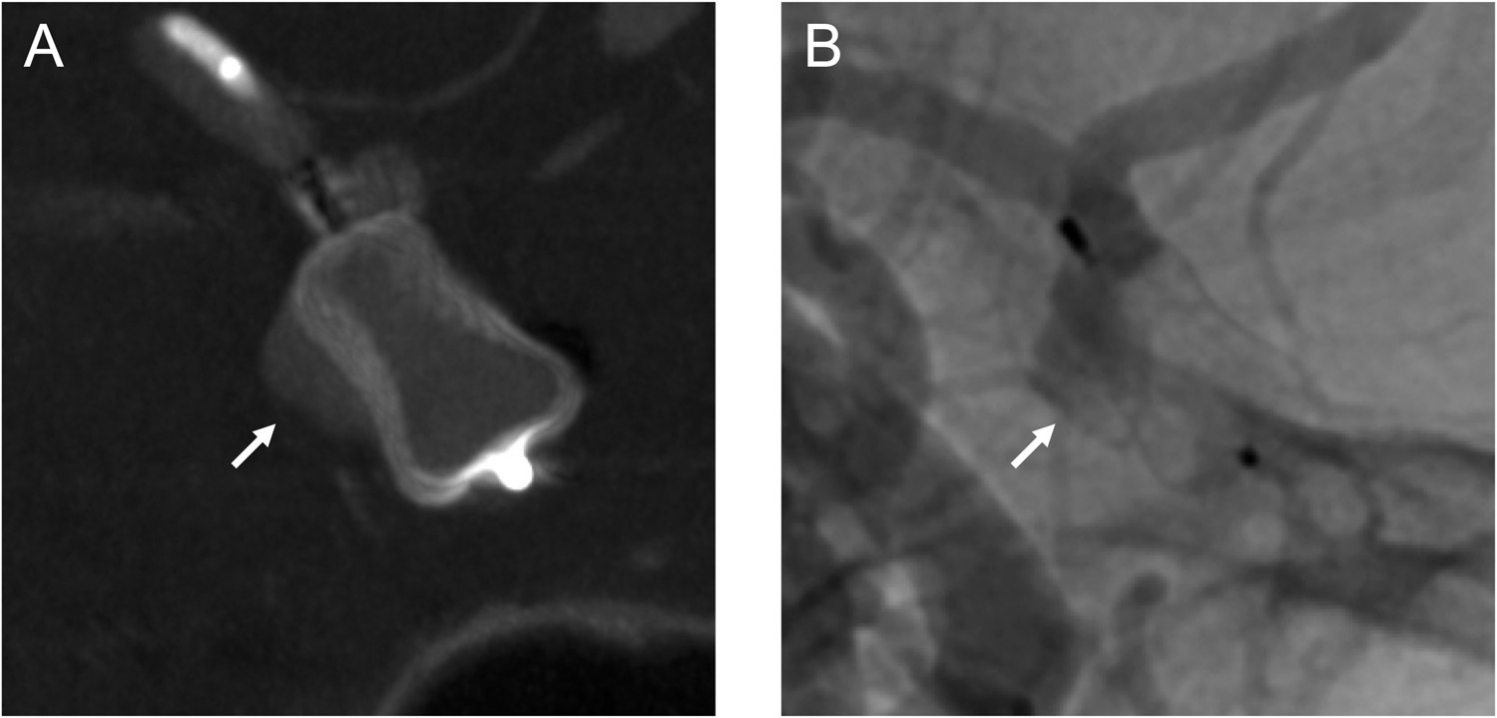
(A) Cone-beam computed tomography and (B) angiography demonstrating deployment of the Woven EndoBridge device within the aneurysm sac, avoiding the area compressing the optic nerve (arrows).

**Fig. 4:**
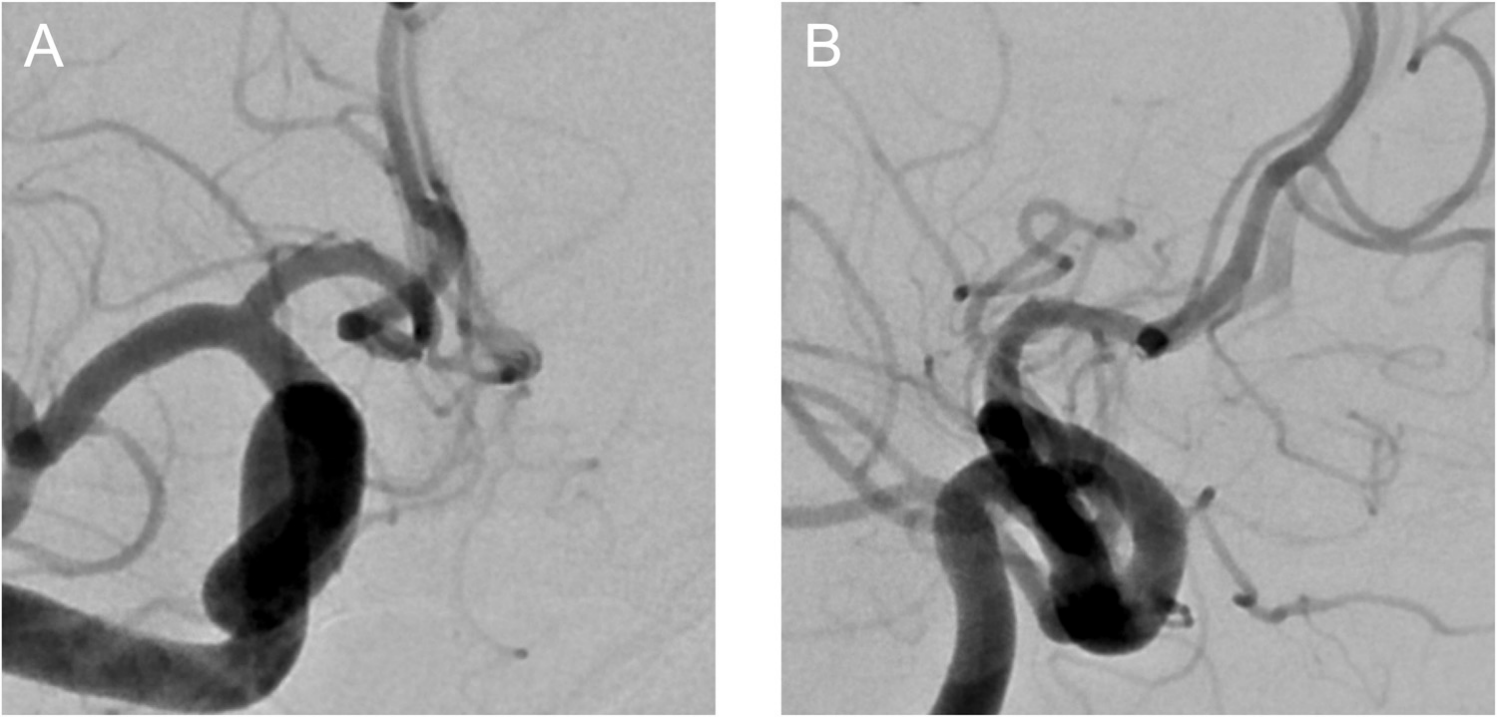
Six-month follow-up angiograms demonstrating complete occlusion of the aneurysm. (A) Anteroposterior view and (B) Lateral view.

A 72-year-old woman was incidentally found to have an unruptured ACoA aneurysm on magnetic resonance imaging and magnetic resonance angiography. Digital subtraction angiography revealed the aneurysm at the right anterior cerebral artery A1-A2 junction, with a maximum diameter of 8.01 mm (Fig. 1). Although the patient reported no subjective visual symptoms, imaging analysis using constructive interference in steady-state magnetic resonance imaging and its fusion with angiography demonstrated compression of the right optic nerve from the medial superior aspect by the aneurysm (Fig. 2). An ophthalmologic evaluation was therefore requested, revealing an abnormal visual field in the inferior lateral quadrant of the right eye, consistent with the imaging findings. The patient opted for endovascular treatment of the aneurysm. Endosaccular embolization using the WEB device was performed under general anesthesia. The device was deployed within the aneurysm sac, taking care to prevent its expansion toward the area compressing the optic nerve (Fig. 3). The postoperative course was uneventful, and the patient was discharged without complications. At the 1-month postoperative visual field examination, her visual field abnormality had improved. No impact was found on her visual acuity or color vision before and after treatment. A 6-month follow-up angiogram confirmed complete occlusion of the aneurysm (Fig. 4).

## Discussion

This case of WEB treatment for an ACoA aneurysm with optic neuropathy resulted in postoperative improvement. ACoA (or anterior cerebral artery) aneurysms can cause compressive optic neuropathy, leading to visual field defects and decreased visual acuity [[3], [4], [5], [6], [7], [8], [9]]. Ruptured ACoA aneurysms are known to cause optic neuropathy even when small, with arterial blood flow and/or hematoma likely contributing factors [3]. Unruptured ACoA aneurysms rarely result in optic neuropathy, though cases have been reported in large or giant aneurysms and in partially thrombosed aneurysms [[4], [5], [6], [7], [8]]. Very rarely, a small aneurysm has been implicated [9]. In terms of treatment, previous reports have described craniotomy clipping and endovascular coiling [[3], [4], [5], [6], [7], [8], [9]]. However, to the best of our knowledge, no previous reports have documented WEB treatment for ACoA aneurysms presenting with optic neuropathy. In this case, WEB treatment proved effective. We considered 2 possible mechanisms for the observed improvement: first, the disappearance of pulsation due to aneurysm thrombosis, and second, a reduction in mass effect (the WEB device does not expand to fill the entire aneurysm). The structural design of the WEB device prevents its mesh from contacting protruding areas such as blebs. Therefore, if the protruding portion of the aneurysm compresses the optic nerve, as in our case, the WEB device may help reduce the mass effect (Fig. 3). Alternative endovascular approaches (eg, coiling ± stent assistance) generally involve coils filling the entire aneurysm, making it difficult to reduce mass effect and optic nerve compression. However, these remain speculative, and further studies are needed to clarify the accurate mechanisms. This case report has several limitations that must be considered: the small number of cases (a single case report from 1 institution), the mild severity of preoperative optic neuropathy, and potential challenges in controlling WEB deployment. Further investigation with larger case series will be necessary.

## Conclusion

We presented a case of WEB treatment for ACoA aneurysm presenting with compressive optic neuropathy. This report demonstrates safe aneurysm occlusion and visual improvement with WEB deployment. WEB treatment may represent a useful therapeutic option for aneurysms associated with optic neuropathy.

## Ethical statement

All procedures performed in studies involving human participants were in accordance with the ethical standards of the institution and/or national research committee and with the 1964 Helsinki declaration and its later amendments or comparable ethical standards. The study was approved by the Ethics Committee of Nakamura Memorial Hospital (No. 2025102901).

## CRediT author statement

**Hideki Endo:** Conceptualization, Methodology, Validation, Formal analysis, Investigation, Resources, Data curation, Writing—original draft, Writing—review and editing, Visualization, Supervision, Project administration; **Yasuyuki Tatsuta:** Validation, Resources, Writing—review and editing; **Koichiro Shindo:** Validation, Writing—review and editing; **Daishi Yamaguchi:** Validation, Resources; **Suguru Sakurai:** Validation, Resources; **Yohei Yamaguchi:** Validation, Resources; **Ryunosuke Yoshihara:** Validation; **Daigo Goto:** Validation; **Masahiro Okuma:** Validation; **Tomoaki Ishizuka:** Validation; **Tatsuya Ogino:** Validation, Resources; **Hirohiko Nakamura:** Supervision.

## Patient consent

Written informed consent was obtained from the patient.
